# Are live lectures a discontinued model? A survey on the influence of synchronous online lecturing on the perception of teaching and assessment outcome

**DOI:** 10.3205/zma001632

**Published:** 2023-06-15

**Authors:** Jannik Osten, Victoria Behrens, Sadie Behrens, Andreas Herrler, Tim Clarner

**Affiliations:** 1 RWTH Aachen, Institute of Neuroanatomy, Aachen, Germany; 2 Maastricht University, FHML, Anatomy & Embryology, Maastricht, The Netherlands; 3 Rostock University Medical Center, Institute of Anatomy, Rostock, Germany

**Keywords:** medical education, online lecture, histology

## Abstract

**Objectives:**

In the early phase of their studies, students are confronted with a number of teaching and learning methods they are usually not familiar with. Beyond, learning in a university environment requires a high degree of self-organization. Thus, the transition from learning in a school environment to university can be challenging for students and associated with adjustment difficulties.

We hypothesized that synchronous online lecturing might be able to serve as a thematic superstructure and a curricular guide that can positively influence course perception, motivation and exam outcome.

**Methods:**

We investigated this hypothesis in a retrospective approach by comparing results from histology exams (2020 n=411, 2021 n= 423) and questionnaires for course evaluation received from medical and dentistry second semester students of the RWTH Aachen University, Germany, in 2020 (n=113 questionnaire participants) and 2021 (n=106 questionnaire participants). While in 2020, due to the Corona Pandemic, no synchronous online lectures were held, these were reintroduced in 2021.

**Results:**

Our results show several differences in between the two study cohorts. Most important findings include a significantly (p<0.001) lower number of students that failed to pass or withdrew from the exam in 2021, an increased motivation to deal with the learning content (p<0.001) and a higher perceived quality of the study materials (p<0.001) in 2021.

**Conclusion:**

Our study indicates that synchronous online lectures can be an important tool to help students to accustom to new learning environments and to structure private study. Further studies will now have to show whether live (online) lectures can have the same significance during clinical training.

## Zusammenfassung

**Hypothese:**

In der Frühphase ihres Studiums werden Studierende mit einer Vielzahl von Lehr- und Lernmethoden konfrontiert, die ihnen in der Regel nicht vertraut sind. Darüber hinaus erfordert das Lernen in einem universitären Umfeld ein hohes Maß an Selbstorganisation. Somit kann der Übergang vom Lernen in einer Schulumgebung zur Universität für Studierende herausfordernd und mit Anpassungsschwierigkeiten verbunden sein.

Wir stellten die Hypothese auf, dass synchrone Online-Vorlesungen als thematischer Überbau und als curricularer Leitfaden dienen können, der die Kurswahrnehmung, die Motivation und die Prüfungsergebnisse positiv beeinflussen kann.

**Material und Methode:**

Wir untersuchten diese Hypothese in einem retrospektiven Ansatz, indem wir die Ergebnisse von Histologieprüfungen (2020 n=411, 2021 n= 423) und Fragebögen zur Lehrveranstaltungsevaluation verglichen, die wir von Medizin- und Zahnmedizinstudierenden des zweiten Semesters der RWTH Aachen, Deutschland, in den Jahren 2020 (n=113 Fragebogenteilnehmer) und 2021 (n=106 Fragebogenteilnehmer) erhielten. Während im Jahr 2020 aufgrund der Corona-Pandemie keine synchronen Online-Vorlesungen stattfanden, wurden diese im Jahr 2021 wieder eingeführt.

**Ergebnisse:**

Unsere Ergebnisse zeigen mehrere Unterschiede zwischen den beiden Studienkohorten. Zu den wichtigsten Erkenntnissen gehören eine signifikant (p<0,001) geringere Anzahl von Studierenden, die die Prüfung im Jahr 2021 nicht bestanden oder sie abgebrochen haben, eine höhere Motivation, sich mit den Lerninhalten auseinanderzusetzen (p<0,001) und eine höhere wahrgenommene Qualität der Studienmaterialien (p<0,001).

**Schlussfolgerung:**

Unsere Studie deutet darauf hin, dass synchrone Online-Vorlesungen ein wichtiges Instrument sein können, um den Studierenden zu helfen, sich an neue Lernumgebungen zu gewöhnen und das Selbststudium zu strukturieren. Weitere Studien werden nun zeigen müssen, ob Live-(Online-)Vorlesungen während der klinischen Ausbildung die gleiche Bedeutung haben können.

## 1. Introduction

Academic teaching is subject to constant change and the idea of what is “good teaching” is highly variable in between individuals, subjects and (teaching) cultures. Historically, oral live lectures are a popular tool of imparting knowledge [1]. Reflecting changes in the zeitgeist, these “chalk and talk” lectures widely have changed into a more dynamic, interactive format [2], [3].With respect to teaching in the medical field, didactic lectures have been the primary teaching method for a long time, especially in the preclinical years [4]. However, student attendance at lectures has been steadily declining over time [5]. The reasons for this are manifold, for example, a shift in educational approaches to emphasize more student-centered, self-directed learning or the availability of online lectures might decrease the necessity to attend a lecture live and/or in presence [6], [7]. Indeed, growing consensus is made telling us that humans learn best when they are active and engaged, when the material to be learned is meaningful (not disjointed), and when it occurs in a socially interactive context that is iterative and fun [8]. 

When it comes to “active learning”, however, there is no clear definition of what is meant by this term [9], thus raising the questions if there are circumstances under which students can actively learn during lecture. Lombardi and colleagues currently proposed a construction-of-understanding ecosystem for active learning in which they highlight the importance of a variety of potential flows of meaning in active-learning settings. In this work they challenge the definition of active learning as the antithesis to lecture.

There is general consensus among anatomists that microscopic anatomy is a crucial part of pre-clinical education. A solid histological knowledge is the basis for functional understanding and many diagnostic skills. The way microscopic anatomy is taught differs in between universities and so does the extent to which problem-solving and self-learning is integrated [10]. However, in general students learn microscopic anatomy through the three mechanisms “memorization”, “comprehension” and “visualization”. It is mainly up to the university and the individual teacher to create the right balance of these strategies [11]. The combination of interactive teaching methods in addition to the classic lecture thus might be an interesting way to promote student motivation and interest [12], [13]. Furthermore, the combination of online elements with classical face-to-face anatomy teaching increases the students' learning success and improves self-assessment [14]. 

The transition from school to university learning environment can be challenging [15] and it takes up to a year for students to shed their school habits and learn to work more independently [16]. It has been stated that especially first year students profit from lectures and lecture recordings [17]. Furthermore, the way in which first-year students interact with the learning environment seems to be more important for their success than the grades achieved during school [18], [19]. 

Despite the above-mentioned changes in the educational culture and curricular reorientation, there are also more mundane reasons why lectures are cancelled, such as a lack of teaching staff or – in our case – the short-notice, drastic restrictions due to the Corona Pandemic. 

We used this unintentionally restricted teaching situation to investigate whether classical lectures are still a relevant method to complement self-oriented and autonomous learning approaches or whether they are – indeed – a discontinued model.

In our study, we aimed to address the question whether the absence of live (online) lectures has an impact on overall students’ course perceptions and performance in microscopic anatomy in both human medicine and dentistry students of the second semester. In 2020, due to pandemic-related short-notice regulatory requirements at the RWTH Aachen University, the 2nd semester microscopic anatomy course was held without accompanying live lecture. The focus was on an active self-directed teaching format using online synchronous question and answer sessions (Q&A). In the following year 2021, synchronous online lectures were possible again due to the integration of new software and docent training. Accordingly, the online Q&A sessions were not conducted anymore. Note that the overall course structure, learning material and setup of test remained identical in both investigated years.

In a retro-perspective approach, the students were asked about their perception of the course and the quality of the teaching content, using a Likert-Scale and Multiple-Choice-Based questionnaire. In addition, we compared exam results of both study cohorts to investigate whether significant differences could be observed in teaching/ learning efficacy.

## 2. Materials and methods

### 2.1. Ethical approval

The ethics committee of the medical faculty of RWTH Aachen University has stated that there are no ethical or professional concerns about the research project (EK 141/22). 

The participants gave informed consent. 

### 2.2. Course structure

The 2nd semester course of microscopic anatomy took place in 2020 for human medicine and dentistry students at the Medical School of Aachen University, Germany. Due to the pandemic related restrictions, no live lectures were held in this year. Instead, students asked questions via email and in two Q&A sessions via Zoom (Zoom Video Communications, San José, USA). Different course materials were made available to the students composed of self-study and interactive learning methods:

1. lecture slides (PDF),
2. script on microscopy and histology (PDF),
3. virtual online microscopy program (Aperio eSlideManager, Leica Biosystems, Nussloch Germany),
4. a digital online practical, in the form of video-based explanations of exemplary histological sections,
5. the histology textbook.

With these materials, students were supposed to learn in a merely self-structured way. 

In the following year of 2021 a synchronous online lecture with a total of 16 lessons of 45 minutes each was held online via Zoom. Digitally retrievable recordings of the lecture were made available. All other course contents as mentioned above remained the same with the exception of the omitted Q&A session (see table 1 (Tab. 1)).

### 2.3. Questionnaire and survey

113 students took part in the study in 2020 (n=90 human medicine, n=23 dentistry). In 2021, a total of 106 students were surveyed (n=82 human medicine, n=24 dentistry). Survey took place online using Microsoft Forms (Microsoft, Redmond, USA). The anonymous questionnaire was provided exclusively to the course participants. No distinction was made between age, gender or other personal attributes. The survey took place immediately after completion of the course but before the final exam in order to exclude a possible influence of a positive or negative exam result on overall course perception. Surveys answered after the exam were excluded from the evaluation. The questionnaire consisted of 18 questions in German language (see table 2 (Tab. 2)), asking for the study subject (human medicine or dentistry), two multiple-choice questions, 12 Likert scale based questions and three free text questions. The grading of the Likert scale (question No. 4-13,15) was as follows: 1: do not agree at all, 2: do not agree, 3: undecided, 4: agree and 5: completely agree and in case of question 14: 1: very low quality, 2: low quality, 3: medium quality, 4: high quality and 5: very high quality. The three free text questions asked for overall aspects of the course that were particularly liked, not liked at all and what would be possible suggestions for improvement. In order to highlight the most important elements, the free text answers were grouped into topic areas for better comparability by screening the answers for key words that were mentioned more than once. A statistical evaluation beyond the normal descriptive statistics was not practical due to the free text character of the answers. The full questionnaire can be provided by the authors upon request.

### 2.4. Statistical analysis

We have performed a statistical analysis using IBM SPSS Statistics for Macintosh, Version 28.0. (Armonk, NY: IBM Corp.). The Kolmogorov-Smirnov-Test revealed that the questionnaire answers were not normally distributed, with p<0.05. For this reason, we used a Mann-Whitney U-test for our data analysis and the Pearson r was calculated as well.

The p values were set as *p≤0.05, **p≤0.01 and ***p≤0.001. In the following, the effect size using Pearson r correlation was calculated to classify the strength of the relation. In addition to the data obtained from the questionnaire, we examined the exam results.

## 3. Results

### 3.1. Questionnaire

The proportions of students of human medicine (h) and dentistry (d) at our faculty correspond approximately to an 80%(h)/20%(d) ratio. This ratio was also found in our study. In 2020 the ratio was 80%(h)/20%(d) and in 2021 77%(h)/23%(d).

Regarding the usage (Q. No. 2) and usefulness (Q. No. 3) of the different course materials, the perception of the two cohorts changed from 2020 (no lecture) to 2021 (online lecture). In 2020, the textbook, which was used by 57% of the students, was perceived as most helpful for knowledge generation, ahead of the lecture slides (50% usage) and the digital practical (43% usage). By contrast, in 2021, the lecture slides (72% usage) were considered to be by far the most helpful teaching medium followed by the digital practical (55% usage) and the lecture itself (51% usage). 

**Figure 1:**
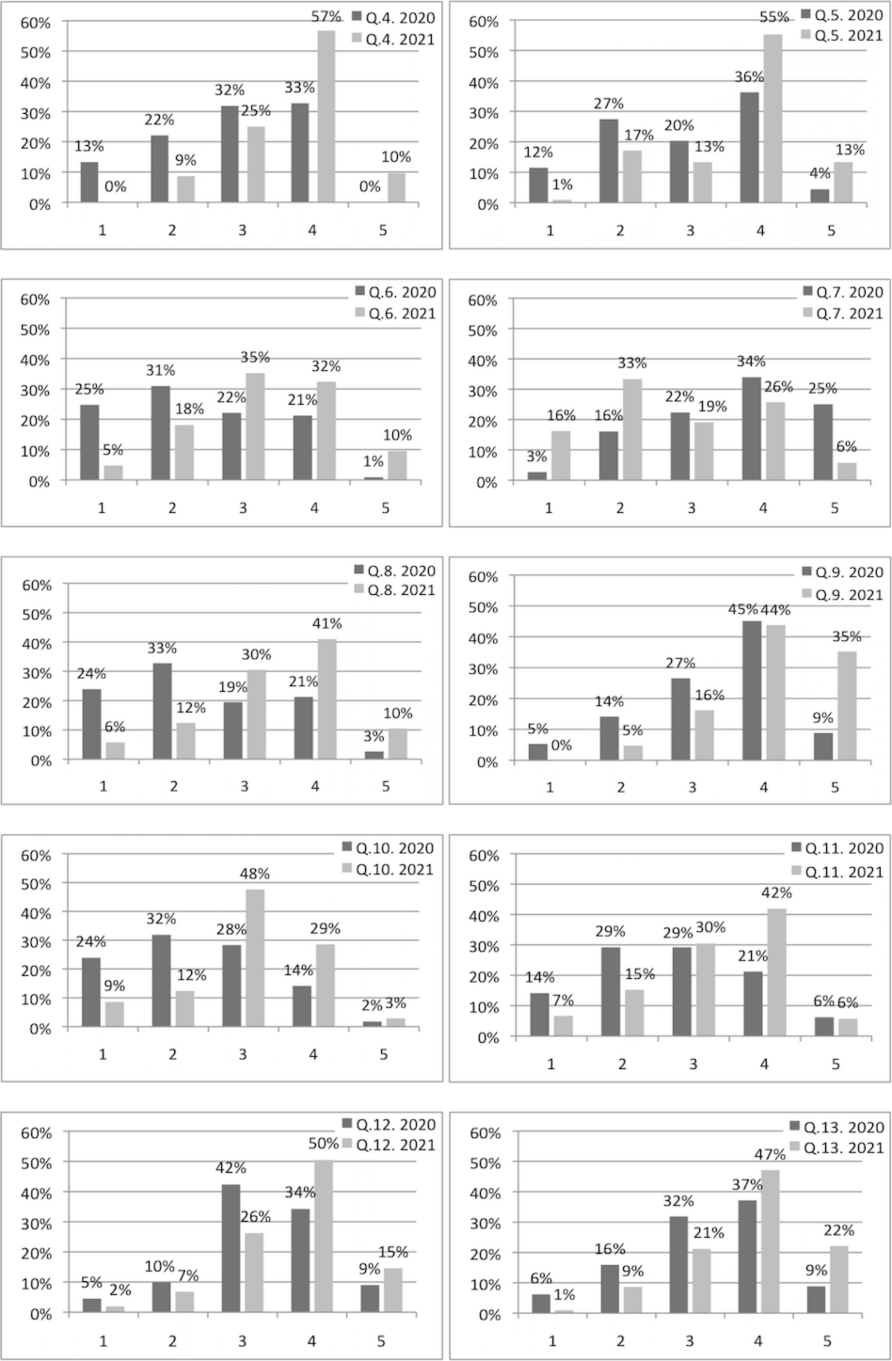
Distribution of Likert Scale answers questions no. 4-13. Likert Scales: 1: do not agree at all, 2: do not agree, 3: undecided, 4: agree and 5: completely agree. The queried content for each above shown question can be found in table 2.

If we look at the Likert scale questions 4-13, we can generally say that we found strong significant differences between the answers of year 2020 and 2021 (see table 3 (Tab. 3) and for quantitative information see figure 1 (Fig. 1)). For better illustration we thematically divide the questions 4-13 into the topics: given curricular structure (Question No. 5,7,11,12), personal structure (Q. No. 4,6), contact person/interaction (Q. No. 13,9), motivation (Q. No. 8) and exam preparation (Q. No. 10). 

In the subject area of the given curricular structure, the design of the course offerings (Q. No. 5) was rated as significantly clearer in 2021. The course objectives (Q. No. 11) were also perceived as more clearly defined in 2021 and students felt that there was a better constructive alignment of objectives and content (Q. No. 12). In line, a predefined learning structure was missed more often in 2020 (Q. No. 7). 

Regarding personal structure, students indicated that they felt significantly better able to acquire the necessary subject matter based on the teaching materials in 2021 (Q. No. 4). Moreover, it was easier for them to develop their own learning structure with the help of the teaching materials (Q. No. 6). In addition, we have also seen a significant change towards higher motivation to deal with the learning content (Q. No. 8).

Furthermore, the students had a stronger feeling in 2021 to always have a contact person to ask questions (Q. No. 9). Additionally, the possibility to interact directly with a lecturer (Q. No. 13) was perceived more positive.

In addition to the improvements described above in the areas of learning structure, motivation and interaction, the feeling of being adequately prepared for the final exam has also improved (Q. No. 10). This subjective feeling can also be objectified and confirmed by evaluating exam results (Section 3.3 Exam results).

Although the students received identical course material in both years, the perceived quality of the materials (Q. No. 14) was rated significantly better in 2021. Interestingly, not only the quality of the materials, but also their suitability for relevant knowledge generation and exam preparation (Q. No. 15) was rated higher in 2021. 

### 3.2. Free text answers

When asked what the students liked most (Q. No. 16) about the 2020 course, the most common responses were the digital practical course (31.4%) and virtual microscopy (15.7%). 

In the following year 2021, the lecture (26.0%), followed by the virtual microscopy (22.0%), as well as the digital practical course (20.0%), were the elements that the students liked most. 

In 2020, the most common criticism was the difficulty to independently weight certain topics, which was stated by 25.6% of the students (Q. No. 17). Moreover, 18.6% of the students missed an overarching learning structure in the course of 2020. In contrast, 20.9% of the students cited the large amount of learning content as biggest criticism in 2021.

With regard to possible suggestions for improvement (Q. No. 18), by far the most frequent response for 2020 was the wish for an accompanying lecture to be held again, expressed by 37.7% of respondents. For 2021, no overarching themes with a central tendency could be formed for the possible suggestions for improvement. 

### 3.3. Exam results

**Figure 2:**
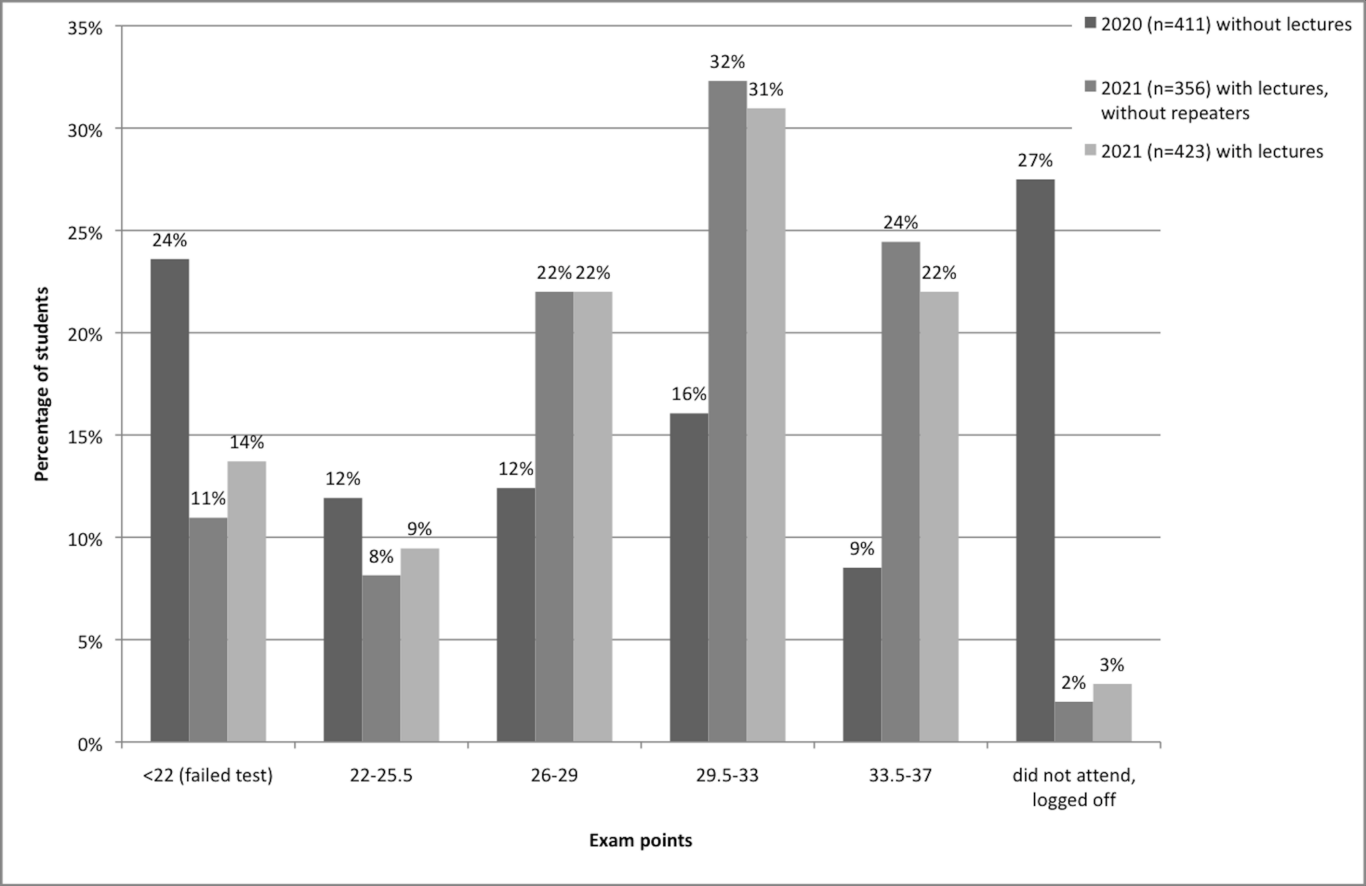
Exam results. Overview of exam results for the course years 2020, 2021 and 2021 without repeaters from 2020.

To evaluate whether this self-evaluation indeed correlates with exam results, we compared both years (see figure 2 (Fig. 2)). In both years, a maximum of 37 points could be reached in the exam. As shown in figure 2 (Fig. 2), the points achieved by the students for the respective exams ranged from 4 to 37. While in 2020, 24% of students failed to pass the exam, this number declined to 14% in 2021. Since repeaters from 2020 might be a possible bias in the 2021 cohort due to more practice and longer learning time, these were additionally excluded from the data. However, by excluding repeaters, percentage of failed exams decreases to 11%. While the grade point average achieved in 2020 was 24.4 (SD±8.0), in 2021 it was 28.4 (SD±6.4) and 29.0 (SD±5,9) without course repeaters. This observation was also confirmed by the statistical analysis using the Mann-Whitney U test, which showed a highly significant difference between the results 2020 and 2021(p<0.001, Z=-6.853, r=0.26). In addition, the number of students who withdrew from the exam in advance or did not show up changed noticeably. It fell from 27% in 2020 to only 3% (2% without course repeaters) the following year. 

There was no significant difference (p=0.4994, Z=-0.6755, r=0.02) between the results from students in 2021 calculated with or without the course repeaters.

## 4. Discussion

Based on our results for a preclinical course in microscopic anatomy, we can conclude that students benefit from a synchronous online lecture (year 2021) in many ways. This was reflected by improvements regarding personal motivation to deal with course content, interaction with teachers, the feeling of being adequately prepared for the exam, the perceived course structure and perceived quality of the course content. Furthermore, the exam results were significantly better in 2021. This is in line with the high acceptance of lectures in the preclinical study section [20]. In our opinion, lectures create a necessity and motivation for students to deal with the subject matter in a given time frame. This is comparable to certain subjects being taught at certain times in the school curriculum. This may help to develop learning self-discipline around the lectures as a fixed framework. In courses without direct instruction from a teacher, students who are not accustomed to self-directed learning often have concerns about not covering or missing important content. This can be remedied by proper balance of cooperative course content integrated into the conventional lecture [21]. The observation of students' concern about missing out on important content during self-study is consistent with our research. However, one might want to discuss whether lectures indeed have to be “live” or whether educational videos might cover many of the above-mentioned students expectations and needs as well. Indeed, precast videos might even have some advantages above synchronous online lectures such as availability at all times, the possibility to pause at any time and to combine them with self-assessment tools [22], [23], [24], [25].

Considering the expressed difficulties in weighting certain teaching contents independently, it is interesting to note that a defined catalogue of learning objectives for the course was available to students. However, the personal weighting by a teacher in the direct temporal context of the presented material seems to stimulate students more strongly. Through the lecturer's enthusiasm for a topic, the student can estimate the relevance of the material taught in the lecture [26]. This is also reflected in the suggestions for improvement for 2020, where the students explicitly wish for an accompanying live lecture. Our data suggest that lectures can be a meaningful addition to interactive and self-directed teaching content. However, emphasizing what we believe to be the particular importance of the lecture in a curricular structure should not be seen as an appeal for excessive classical frontal teaching. In our opinion, the insights gained do not speak against the further development or implementation of interactive teaching content into this “classic” lecture. Depending on the topic, there are many ways to make a lecture interactive e.g. “flipped classroom” models [3]. The implementation of interactive subjects as short sessions (10-15 min) within a lecture are highly appreciated by the students as they feel more connected to the subject matter [21].

We would like to point out that students, especially in their first year of study, may have some concerns and adjustment problems when they find themselves in an unfamiliar self-directed learning environment. This might be a possible explanation for the more negative course perception in 2020. In addition, there is a reinforcing relationship between uncertainty and self-handicapping behavior [27], [28], which can lead to poorer exam results, as observed in our study. Besides, it is important for teachers to know what skills are important for the success of an independent learning individual. The prerequisites for being successful as an independent self-learner is the acquisition of a self-learning competence with associated skills. These are independence, a willingness to engage in lifelong learning, an associated spirit of inquiry, and organizational skills to structure academic and personal concerns [29].

Regarding to organizing things better, we observed a significant improvement in the ability to adopt a personal learning structure on the basis of the learning materials in 2021. This is complemented by the statement of the students who missed an overarching learning structure in 2020. In order to structure personal and academic concerns, the familiar lecture method can serve as aid to achieve these goals. In our case, however, this is limited to students in the first preclinical year.

 Additionally, it should be noted that the exam questions for 2020 and 2021 were not identical in order to avoid a possible exchange of information in between the study years. However, structure of the test in 2021 corresponds to those from 2020 with respect to type, learning objectives assessed and difficulty profile. The students in the 2021 course were already accustomed to online-only teaching and to the overall pandemic learning situation. A possible bias by repeaters from 2020 in the 2021 course could not be confirmed since results even improved by excluding the repeaters from the analysis. It would be interesting to, in further studies, investigate whether those repeaters have more problems in acquiring a self-learning competence, especially in a course with online emphasis.

The poorer results in 2020 thus may in part be due to problems in adapting to a purely online teaching curriculum and a general pandemic-related uncertainty. A possible form of habituation to the existing pandemic may also be partly responsible for a more positive course perception in 2021.

However, this limitation applies to all studies conducted in the first and subsequent years of the pandemic, as the influence of such a major impact on daily life and work cannot be eliminated. 

## 5. Conclusions and outlook

We conclude that especially for students in the preclinical section who are not yet used to self-oriented “university-style” learning, the synchronous (online) lecture is NOT a discontinued model. Based on our study and apart from imparting knowledge, the value of the lecture can be seen in providing a thematic superstructure and a curricular guide. The structured learning environment of (online) live lectures leads to a general increase in self-confidence in the students’ abilities. This even means that the quality of other course contents is rated higher, leading to better exam results. The lectures in the first years help the students to develop independent and self-directed learning skills. 

It might now be interesting to ask whether similar conclusions would be reached in the clinical semesters. Does the value of the lecture for the students decrease in the course of time, or does it shift?

## Abbreviations

- Q&A = Question & Answer
- MC = Multiple Choice
- SC = Single Choice
- LS = Likert Scale
- FT = Free Text

## Acknowledgements

We thank Dr. Melanie Simon for scientific advice and fruitful discussions. 

## Author contributions

This study was conceptualized by Jannik Osten, Victoria Behrens, Sadie Behrens, Andreas Herrler and Tim Clarner. The acquisition, analysis and interpretation of data was performed by Jannik Osten. The original draft was written by Jannik Osten and critically revised by all authors. All authors read and approved the final manuscript. Tim Clarner was responsible for the overall supervision of this study.

## Competing interests

The authors declare that they have no competing interests. 

## Figures and Tables

**Table 1:**
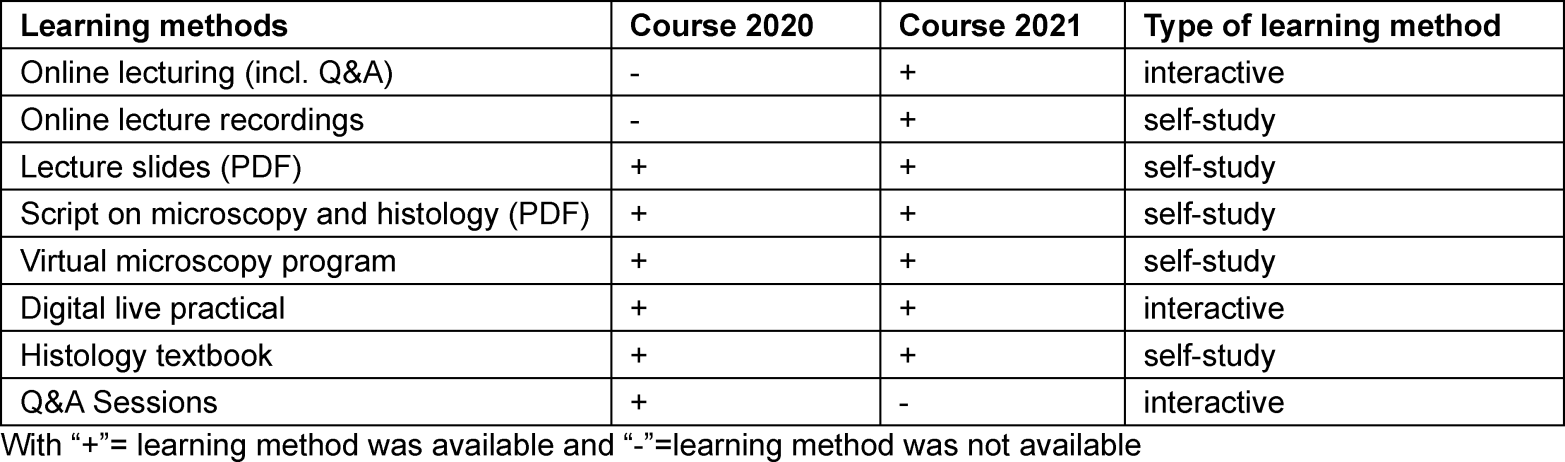
Overview learning methods and thier type for the courses 2020 and 2021

**Table 2:**
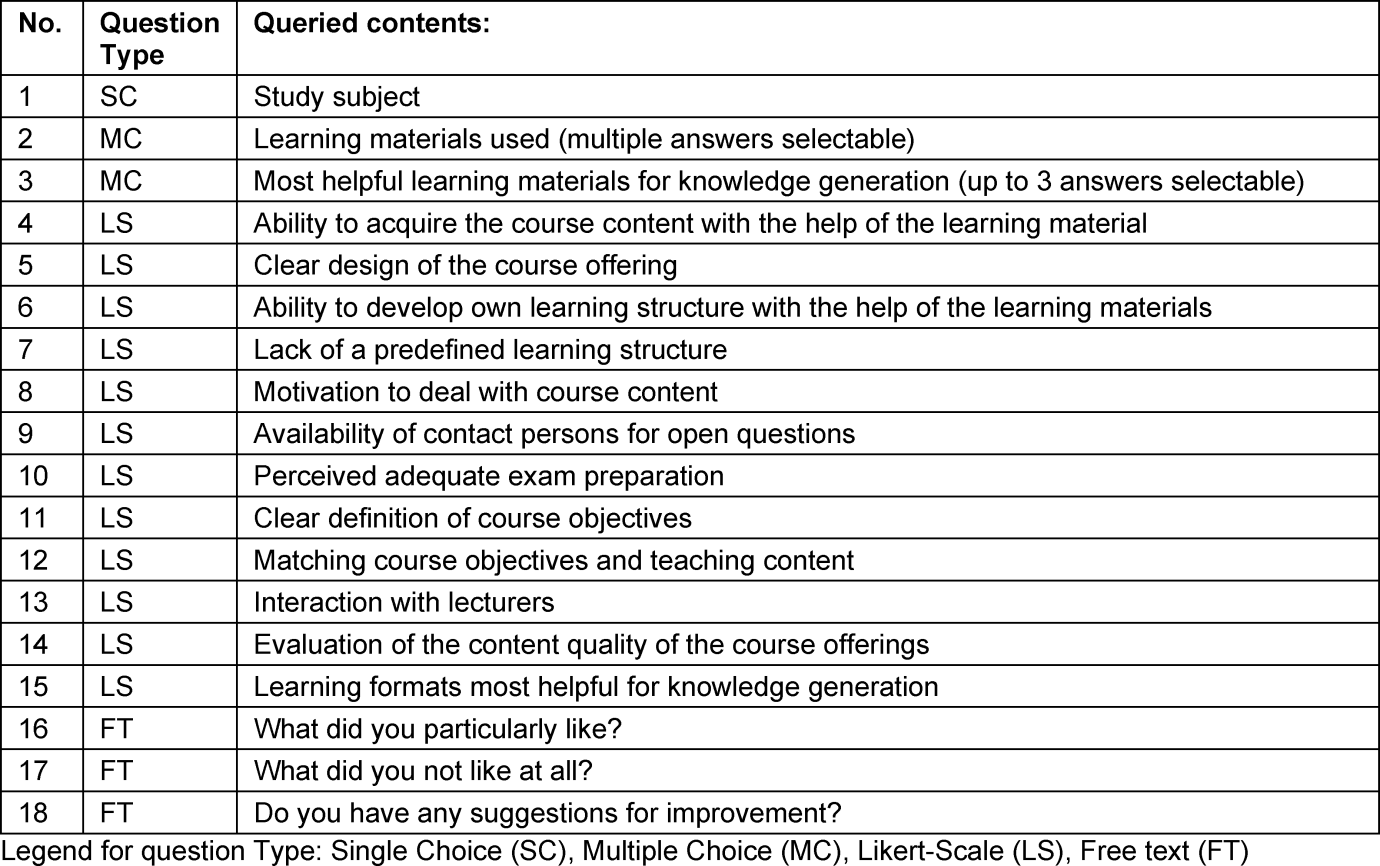
Questionnaire overview

**Table 3:**
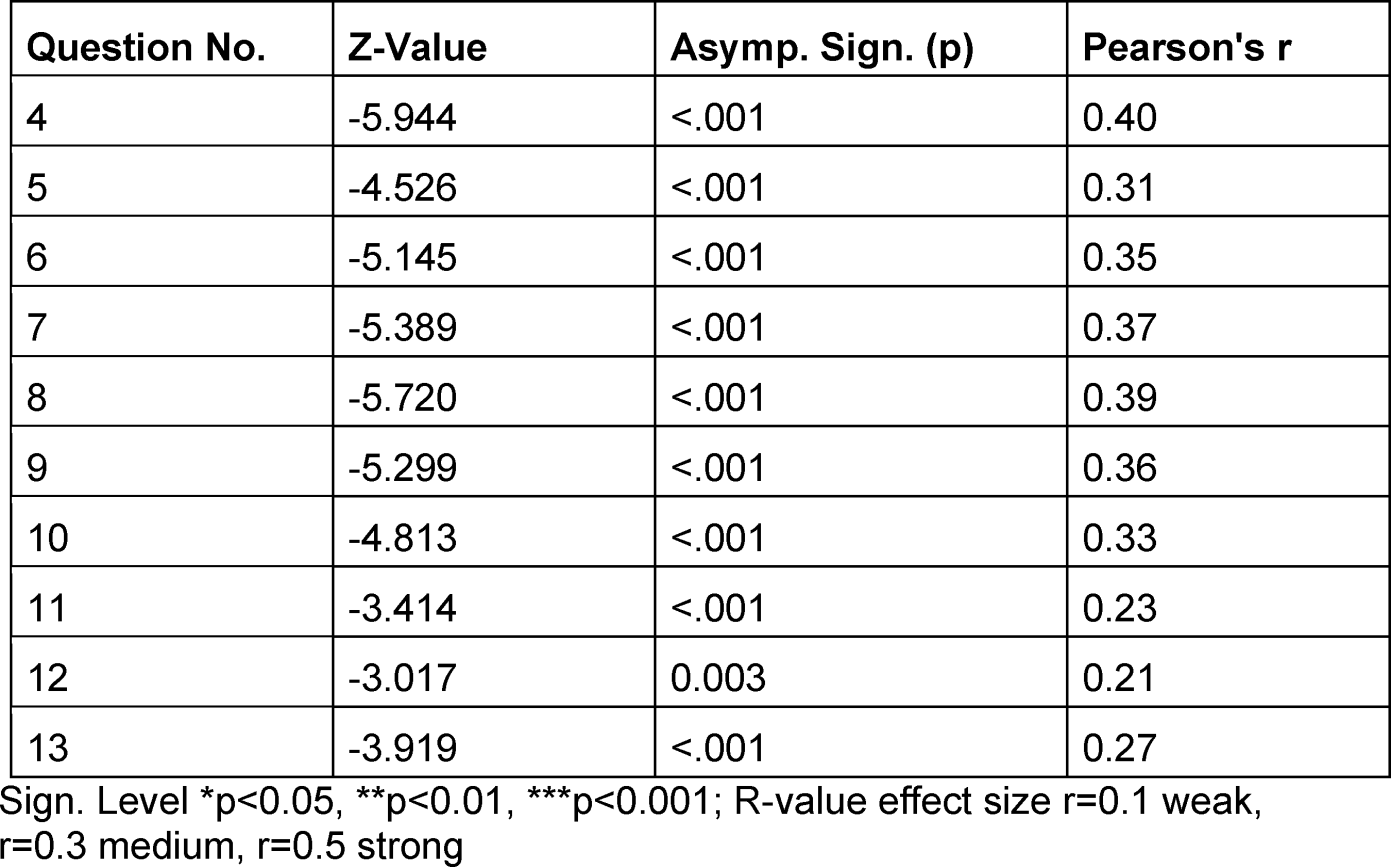
Results of Mann-Whitney U-test, comparing year 2020 and 2021
